# Development of a core set of outcomes in children with severe neuro-disability and feeding tune dependency: a systematic review

**DOI:** 10.1186/1745-6215-16-S3-P9

**Published:** 2015-11-24

**Authors:** Mufiza Z Kapadia, Chrinna Balasingham, Eyal Cohen, Sanjay Mahant, Katherine Nelson, Jonathan L Maguire, Astrid Guttmann, Martin Offringa

**Affiliations:** 1 Toronto Outcomes Research in Child Health (TORCH), SickKids Research Institute, Toronto, Canada; 2 Division of Paediatric Medicine, Department of Paediatrics, Hospital for Sick Children, Toronto, Canada; 3 Institute for Health Policy, Management and Evaluation, University of Toronto, Toronto, Canada; 4 Institute for Clinical Evaluative Sciences, Toronto, Canada; 5 CanChild centre for Childhood Disability Research, Hamilton, Ontario, Canada; 6 Pediatrics Outcomes Research Team, Division of Paediatric Medicine, Department of Paediatrics, The Hospital for Sick Children, Toronto, Ontario, Canada; 7 Paediatric Advanced Care Team, Department of Paediatrics, Hospital for Sick Children, Toronto, Ontario, Canada; 8 Paediatrics, Faculty of Medicine, University of Toronto, Toronto, Canada; 9 Departments of Paediatrics, St. Michael’s Hospital, Toronto, Canada

## Background/Aim

Children with severe neuro-disability are at increased risk of feeding problems resulting in approximately half of such children being undernourished with growth failure. While gastrostomy tube feeding in such patients has been shown to improve weight gain, there is uncertainty to its impact on survival, respiratory complications, parental and child quality of life, cost, and consequently leads to potentially avoidable variability in practice. The issue of lack of standardized outcomes for this population could be addressed through the development of a standardized core outcome set (COS). We aim to develop an evidenced based COS for children 0-18 years with severe neuro-disability and dependent gastrostomy.

## Methods and Results

A systematic review was undertaken to identify all outcome measures used in studies on children with severe neuro-disability dependent on gastrostomy tube feeding. PRISMA guidelines were followed. MEDLINE, EMBASE and Cochrane Register databases were searched from their inception until March 2015. Studies included systematic reviews with/without meta-analyses, randomised controlled trials, and observational studies. After initial screening of titles and abstracts, disagreements on the eligibility of studies were resolved through discussion. Data were extracted on study characteristics, outcomes measured, designated primary and secondary outcome(s), method of measurement, and time points at which they were measured. A thematic content analysis was undertaken to map the outcomes against the OMERACT framework that included four pre-specified outcome “domains”: mortality, pathophysiological manifestations, patient reported outcomes and healthcare utilization. To date, 8725 titles and abstracts are screened. A total of 1460 studies were found eligible. Final results will be presented at the meeting.

## Discussion

Outcomes identified through this systematic review will be used to help stakeholders reach consensus on a COS for research in children with severe neuro-disability and feeding tube dependency which could be used to enhance future research, knowledge synthesis and inform clinical practice.

